# Serum Metabolomics and NF-κB Pathway Analysis Revealed the Antipyretic Mechanism of Ellagic Acid on LPS-Induced Fever in Rabbits

**DOI:** 10.3390/metabo14080407

**Published:** 2024-07-25

**Authors:** Feng-Feng Xie, Li-Ba Xu, Hua Zhu, Xiu-Qi Yu, Lin-Yu Deng, Hui-Zhen Qin, Si Lin

**Affiliations:** 1 Guangxi Key Laboratory of Zhuang and Yao Ethnic Medicine, The Collaborative Innovation Center of Zhuang and Yao Ethnic Medicine, Guangxi Engineering Research Center of Ethnic Medicine Resources and Application, Guangxi University of Chinese Medicine, Nanning 530200, Chinaxuliba15078772841@163.com (L.-B.X.); 15078805315@163.com (X.-Q.Y.); dly813_path@163.com (L.-Y.D.); qinhuizhen2020@stu.gxtcmu.edu.cn (H.-Z.Q.); 18728677865@163.com (S.L.); 2 School of Chemistry and Chemical Engineering, Guangxi MinZu University, Nanning 530006, China; 3 Guangxi Institute for DRUG Control, Nanning 530018, China

**Keywords:** ellagic acid, fever, NF-κB/COX-2, metabolomics, gas chromatography–mass spectroscopy

## Abstract

Fever is one of the most common clinical conditions and is characterized by pyrogenic infection, malignancy, inflammation, and tissue damage, among others. Ellagic acid (EA) can inhibit the expression of related proteins on the pathway by blocking the nuclear factor kappa-B(NF-κB) signaling pathway, inhibit the levels of pro-inflammatory factors interleukin-1β(IL-1β), interleukin-6(IL-6), and tumor necrosis factor-α(TNF-α), increase the level of anti-inflammatory factor IL-10, and effectively alleviate inflammatory symptoms. In addition, EA can also reduce the levels of malondialdehyde(MDA) and nitric oxide(NO) in the body, increase the activities of superoxide dismutase (SOD), glutathione (GSH), and catalase(CAT), scavenge oxidative free radicals, inhibit lipid oxidation, and achieve antipyretic and anti-inflammatory effects. The purpose of this study was to establish the relationship between EA and various inflammatory markers, such as TNF-α, IL-6, IL-1β, prostaglandin E2(PGE_2_), and cyclic adenosine monophosphate(cAMP), and clarify the mechanism of the cyclooxidase-2(COX-2)/NF-κB signaling pathway. Combined with the metabolomics analysis, our study revealed the effects of EA on multiple endogenous biomarkers, reflecting the characteristics of a multi-component, multi-target, and multi-pathway mechanism. Compared to lipopolysaccharide (LPS)- treated animals, subsequent administration of EA significantly lowered the LPS-induced rectal temperature increase (*p* < 0.05 or *p* < 0.01), significantly increased serum SOD and GSH levels (*p* < 0.05 or *p* < 0.01), and significantly decreased serum MDA, IL-1β, IL-6, and TNF-α levels (*p* < 0.05 or *p* < 0.01). In addition, compared to LPS-treated animals, subsequent administration of EA significantly decreased cerebrospinal fluid cAMP and PGE_2_ levels (*p* < 0.05 or *p* < 0.01), significantly decreased cAMP, significantly increased 5-HT levels (*p* < 0.05 or *p* < 0.01), and significantly down-regulated p-NF-κB p65 and COX-2 protein levels in the hypothalamus. Subsequent gas chromatography mass spectrometry(GC-MS) metabolite analysis indicated that 12 differential metabolites were detected in serum isolated 4 h after LPS treatment, and 10 differential metabolites were detected in serum collected 7 h after LPS treatment. Next, Pearson correlation analysis was used to systematically characterize the relationship between the identified metabolites and TNF-α, IL-6, MDA, SOD, PGE_2_, and cAMP. The levels of propionic acid, pyridine, and L-valine were up-regulated by EA, which inhibited the expression of MDA, IL-1β, and TNF-α and increased the activity of GSH. The levels of inositol, urea, and 2-monopalmitin were down-regulated by EA, which inhibited the expression of MDA, IL-1β, and TNF-α, increased the activity of SOD and GSH, reduced the inflammatory response, and alleviated the oxidative stress state. Combined with the results of the metabolic pathway analysis, we suggest that the pathways of the galactose metabolism, synthesis and degradation of ketone bodies, as well as ascorbic acid and aldehyde acid metabolism are closely related to the antipyretic and anti-inflammatory effects of EA. Our study established the relationship between EA and various inflammatory markers, such as TNF-α, IL-6, IL-1β, PGE_2_, and cAMP, and clarified the mechanism of the COX-2/NF-κB signaling pathway. Combined with the metabolomics analysis, our study revealed the effects of EA on multiple endogenous biomarkers, reflecting the characteristics of a multi-component, multi-target, and multi-pathway mechanism.

## 1. Introduction

Fever—defined as an elevated body temperature above 37.5 °C—is one of the most common clinical conditions, with a high global mortality due to, among others, pyrogenic infection, malignancy, inflammation, and tissue damage [1], while, equally, being a host defense response against pathogen and/or microorganism invasion, it is generally characterized by body discomfort and adverse effects on the normal function of various body organs [2,3]. High-degree fever approaches the upper limit compatible with human life, making it a dangerous and potentially deadly condition. In fact, an escalating body temperature leads to increased tissue catabolism, dehydration, and organ failure, all of which ultimately promote the advancement of the underlying disease state [4]. Additionally, fever may cause brain cell damage, resulting in muscle twitch, coma, or death [5].

The development of fever involves multiple nerval routes and pro-inflammatory mediators, such as interleukins (ILs), tumor necrosis factor alpha (TNF-α), superoxide dismutase (SOD), glutathione (GSH), malonyldialdehyde (MDA), cyclic adenylmonophosphate (cAMP), prostaglandin E_2_ (PGE_2_), and 5-hydroxytryptamine (5-HT) [6]. In addition, fever is characterized by the activation of multiple pathways centered on nuclear factor kappa-B (NF-κB), p38 MAPK, JNK, TLR4, and cyclooxygenase-2 (COX-2), respectively [7]. Considering that multiple pyrogen types stimulate NF-κB activation, it is generally recognized as the main underlying pathway involved in fever induction, resulting in the high-level secretion of cytokines and free radicals [8]. Currently, non-steroidal anti-inflammatory drugs (NSAIDs), such as aspirin, paracetamol, ibuprofen, and analgin, are generally prescribed to quickly reduce high body temperature [9]. While these compounds block the synthesis of prostaglandins within the hypothalamus through selective COX-2 inhibition, these drugs are equally characterized by selective efficacy or potential (toxic) side effects upon chronic usage of exposure to high dosages, resulting in an increased risk for the development of gastrointestinal ulceration, neutropenia, acute kidney failure, hypertension, allergic reactions, skin toxicity, liver failure, and other bleeding disorders [6]. Therefore, there is an urgent need for new antipyretic molecules with a high efficacy and safety profile.

Metabolomics is a new discipline able to qualitatively and quantitatively analyze the presence of all low-molecular-weight metabolites within an organism or cell [10]. It is a widely used method for the evaluation and screening of drugs through the analysis of the overall composition of the organism as well as the quality evaluation, efficacy screening, and clinical compatibility of, for example, Chinese medicinal materials [11,12,13,14]. Of note, a wide variety of tissue types were previously used to analyze the antipyretic effect of drugs by metabolomics, such as serum [15], plasma [16], brain tissue [17], liver tissue [18], and urine [19].

Considering that serum and plasma contain basic nutrients, hormones, various growth factors, binding proteins, and other substances, these metabolites are often the first sites to be reached by drugs. Of those, inflammatory factors, such as IL-1, IL-1β, IL-6, and TNF-α, are most easily detected in serum and plasma. Currently, chromatographic mass spectrometry approaches such as UPLC-MS and GC-MS are the most popular methods used for metabolomics analyses of metabolic diseases, clinical diagnosis, drug targets, and disease mechanism research [20,21,22].

The endotoxin lipopolysaccharide (LPS)—a strong pro-inflammatory agent able to initiate the inflammatory process through the induction of non-immune cells, which potentially might result in fatal tissues dysfunction—acts as a fever-inducing agent that is typically used to initiate fever in experimental disease models [1,23]. Among the multiple animal models of fever previously described, the LPS-induced fever model in rabbits is the most widely used as rabbits develop fever more easily and have a more docile nature compared to, for example, rats. In general, the innate immune response induced by LPS exposure closely resembles the inflammation-mediated body injury process at the onset of fever. Recent studies indicated that LPS activates both the NF-κB and COX-2 signaling cascades [24], resulting in the release of numerous inflammatory mediators and cytokines, such as ILs, TNF-α, and superoxide anion [25,26,27,28].

Ellagic acid (EA)—a phenolic compound isolated from pomegranate with a relative molecular weight of 302.19 Dalton and molecular formula C_14_H_6_O_8_—has a wide range of biological activities, including antioxidant, anticancer, anti-inflammatory, and antimicrobial functions [29]. Numerous experimental studies reported on the antioxidant and anti-inflammatory properties of EA and its effectiveness in treating acute inflammation, alcoholic liver, airway inflammation, and colitis [30,31,32,33]. Moreover, the oral administration of EA is safe and harmless, with a low degree of toxicity [34].

Based on these characteristics, EA has the potential to be used as an anti-tumor or anti-inflammatory agent. Previous studies indicated that EA suppresses the NF-κB and COX-2 pathways and, as such, limits induction of TNF-α [35,36]. EA can reduce the levels of NO, MDA, IL-1β, and TNF-α, inhibit the expression of COX-2 and NF-κB, and induce the production of GSH and IL-10, which has a protective effect on carrageenan-induced acute inflammation in mice [30]. Moreover, EA was shown to possess nephroprotective effects in a rodent model with lead-induced toxicity through its ability to suppress the NF-κB and COX-2 pathways [37] and subsequent inhibition of TNF-α, IL-6, and COX-2 [38].

In this study, we sought to investigate whether orally administrated EA affects LPS-induced fever in rabbits and to evaluate whether the effect is mediated by the inhibition of the NF-κB and COX-2 pathways. For this, we analyzed the levels of MDA, SOD, GSH, TNF-α, IL-1β, and IL-6 in serum, the levels of PGE_2_ and cAMP in cerebrospinal fluid, and the levels of 5-HT, cAMP in the hypothalamus with enzyme linked immunosorbent assay(ELISA), and the levels of p-NF-κB p65 and COX-2 in the hypothalamus with Western blot. In addition, we performed untargeted metabolomics research on a GC-MS platform to explore biomarkers of LPS-induced hyperthermia in rabbit serum. Metabolomics of the serum samples identified 12 and 10 metabolites, which were affected 4 h and 7 h after LPS exposure, respectively. Interestingly, the levels of seven and six of those identified metabolites were normalized by EA at 4 h and 7 h after LPS exposure, respectively. Taken together, these results provide more evidence to better understand the underlying antipyretic mechanism of EA.

## 2. Materials and Methods

### 2.1. Reagents and Chemicals

Ellagic acid (EA, Batch No. G26J11L119602) was purchased from Shanghai yuanye Bio-Technology Co., Ltd., Shanghai, China. Lipopolysaccharides (LPS, Batch No. 0000081275) were purchased from Sigma Aldrich, MO, USA. Ibuprofen (IB, Batch No. 210313133) was purchased from Shanghai Qiangsheng Pharmaceutical Co., Ltd, Shanghai, China. The assay kits for bicinchoninic acid (BCA, Batch No. 20210727) protein assay were obtained from Nanjing Jiancheng Bioengineering Institute (Nanjing, China). Superoxide dismutase (SOD, Batch No. 20210728) was obtained from Nanjing Jiancheng Bioengineering Institute (Nanjing, China). Malondialdehyde (MDA, Batch No. 20210727) was obtained from Nanjing Jiancheng Bioengineering Institute (Nanjing, China). Glutathione (GSH, Batch No. 20210726) was obtained from Nanjing Jiancheng Bioengineering Institute (Nanjing, China). The rabbit Interleukin-1β (IL-1β, Batch No. I05015518) was purchased from Wuhan Huamei Bioengineering Co., Ltd., Wuhan, China. IL-6 (Batch No. I09015519) was purchased from Wuhan Huamei Bioengineering Co., Ltd., Wuhan, China. Tumor necrosis factor-alpha (TNF-α, Batch No. I04015517) ELISA kits were purchased from Wuhan Huamei Bioengineering Co., Ltd., Wuhan, China. The rabbit cyclic adenosine monophosphate (cAMP, Batch No. 07/2021) was purchased from Shanghai JONLN Reagent Co., Ltd., Shanghai, China. 5-hydroxytryptamine (5-HT) was purchased from Shanghai JONLN Reagent Co., Ltd., Shanghai, China. Prostaglandin E_2_ (PGE_2_) was purchased from Shanghai JONLN Reagent Co., Ltd., Shanghai, China. Further, 99%BSTFA + 1% TMCS (Batch No. N802315, Macklin) was purchased from Shanghai Macklin Biochemical Technology Co., Ltd., Shanghai, China. All other reagents and solvents were of analytical grade and used directly.

### 2.2. Experimental Design

Rabbits (New Zealand, normal class, male, body weight 1.7–2.2 kg) were purchased from Guangxi Guangde Technology Co., Ltd., (Nanning, China, license number: SCXK (Gui) 2017-0001). Rabbits were housed in animal standard houses maintained at a temperature of 23–25 °C and a relative humidity of 40~70%, 12 h/12 h light–dark cycle with free access to standard chow and water. Before the start of the experiment, rabbits were acclimatized to animal standard house for seven days and were fasted 24 h before the experiment while given free access to water. Six rabbits were selected for the normal group (NG). The remaining rabbits received lipopolysaccharide (LPS) (i.v., 4 μg/kg (1 mL/kg) to establish rabbit fever model. One hour after LPS administration, the rectal temperature was checked. A rectal temperature increase ranging between 0.8 °C and 3.0 °C was considered successful in establishing the fever model in rabbits [5]. Next, the LPS-treated rabbits were randomly divided into five groups with six rabbits per group: a model group (MG), an ibuprofen-treated group (IBG, 20 mg/kg), and three groups treated with increasing concentrations of ellagic acid (EALG (26 mg/kg); EAMG (52 mg/kg); and EAHG (104 mg/kg)). NG and MG were given as i.g. 0.5% carboxymethylcellulose sodium (0.5% CMC-Na, 5 mL/kg).

### 2.3. Measurement of Rectal Temperature

The rectal temperature of all animals was measured with an intelligent pyrogen meter ZRY-2D (Tianjin Tianda Tianfa Technology Co., Ltd., Tianjin, China). The rectal temperature was measured twice, and the average value was taken as the baseline temperature. After LPS injection, rectal temperature and its change value were checked once at 1, 3, 5, and 7 h post-injection.

### 2.4. Sample Collection

Blood samples were collected into centrifuge tubes at 4 and 7 h. Serum was isolated by refrigerated centrifuge 5810R (Eppendorf German Ltd., Hamburg, German) for 10 min at 3000 r/min at 4 °C and stored at −80 °C in a refrigerator for analysis. After collecting blood samples, rabbits were sacrificed under anesthesia and the cerebrospinal fluid, and hypothalamus was removed and frozen.

### 2.5. Estimation of Oxidative Stress Biomarkers in Serum

SOD activity was determined by colorimetric method and absorbance at 450 nm according to the manufacturer’s instructions. The levels of GSH were determined by colorimetric method and absorbance at 405 nm according to the manufacturer’s instructions. The levels of MDA were determined by thiobarbituric acid reacting substances (TBARS) method and absorbance at 532 nm according to the manufacturer’s instructions.

### 2.6. Quantifcation of Cytokines in Serum

The levels of IL-1β, IL-6, and TNF-α in serum were measured by ELISA kits according to the manufacturer’s instructions.

### 2.7. Quantifcation of cAMP and PGE_2_ in Cerebrospinal Fluid

After collecting cerebrospinal fluid, the supernatants were isolated by refrigerated centrifuge 5810R for 10 min at 3000 r/min at 4 °C and stored at −80 °C in a refrigerator for determining cAMP and PGE_2_ levels.

### 2.8. Quantifcation of cAMP and 5-HT in Hypothalamus

The hypothalamus homogenates (10% *w*/*v*) were prepared. Protein content was determined in the hypothalamus homogenate following the ELISA procedure of the manufacturer’s instructions. The levels of cAMP and 5-HT in hypothalamus were measured by ELISA according to the manufacturer’s instructions.

### 2.9. Western Blotting

The hypothalamus was extracted from ice with Ripa buffer (containing 0.1% phenylmethylsulfonyl fluoride). The samples were then centrifuged at 4 °C and 12,000 r/min for 10 min. Protein concentrations were determined by the bicinchoninic acid (BCA) protein assay (Servicebio, Wuhan, China). The samples were loaded on 10% sodium dodecyl sulfate-polyacrylamide gel electrophoresis (SDS-PAGE) and transferred onto PVDF membranes (Servicebio, China). Membranes were then probed with primary antibodies (COX-2 and p-NF-κB p65 for hypothalamus) overnight at 4 °C. GAPDH was used as a loading control. The membranes were washed with TBST 3 times and incubated with horseradish peroxidase (HRP)-conjugated secondary antibodies (Servicebio, Wuhan, China) (1:5000) for 30 min at room temperature. Membranes were washed again, as above; enhanced chemiluminescence (ECL) detection reagents were added for 1–2 min and immediately exposed to X-ray film. The density of each band was quantified using Labworks (SSLeye alpha1.0).

### 2.10. Detection of Serum Biomarker Levels with GC-MS

At 4 and 7 h after LPS injection, 3 mL of anticoagulant blood (heparin sodium anticoagulation) was taken from the saphenous vein of the hind limb of the rabbit and centrifuged at 3000 rpm for 10 min. Then, 100 μL of serum was placed in a 2 mL microcentrifuge tube, after which 350 μL methanol was added. The sample was centrifuged for 10 min at 12000 rpm, after which 350 μL of supernatant was placed in a 2 mL microcentrifuge tube and nitrogen dried. Then, 80 μL of ammonium methoxylate reagent (dissolved in 20 mg/mL pyridine) was added. The mixture was heated in a water bath at 70 °C for 2 h, and 100 μL BSTFA (containing 1% TMCS) was added to each sample and further heated in a water bath at 70 °C for 1 h. After cooling to room temperature, 100 μL was centrifuged at 12,000 rpm for 10 min, and 100 μL was taken for detection. Serum samples were measured by GC-MS (7890B-5977B, Agilent Technologies, CA, USA) with the following detection conditions: DB-1701 chromatographic column (30 m × 320 μm × 0.25 μm), carrier gas was high-purity helium, volume flow rate was 2 mL/min, split mode (5:1), injection volume was 1 μL. The temperature programming conditions were as follows: initial temperature was 60 °C for 1 min, then it was increased to 260 °C at 8 °C/min for 2 min, and finally it was increased to 280 °C at 5 °C/min for 5 min. EI ionization collision energy is 70 eV. The ion source temperature was 230 °C, the interface temperature was 280 °C, and the quadrupole temperature was 150 °C. The scanning range of the full scanning mode was *m*/*z*:50~500.

### 2.11. Statistical Analysis

**Figure 1:**
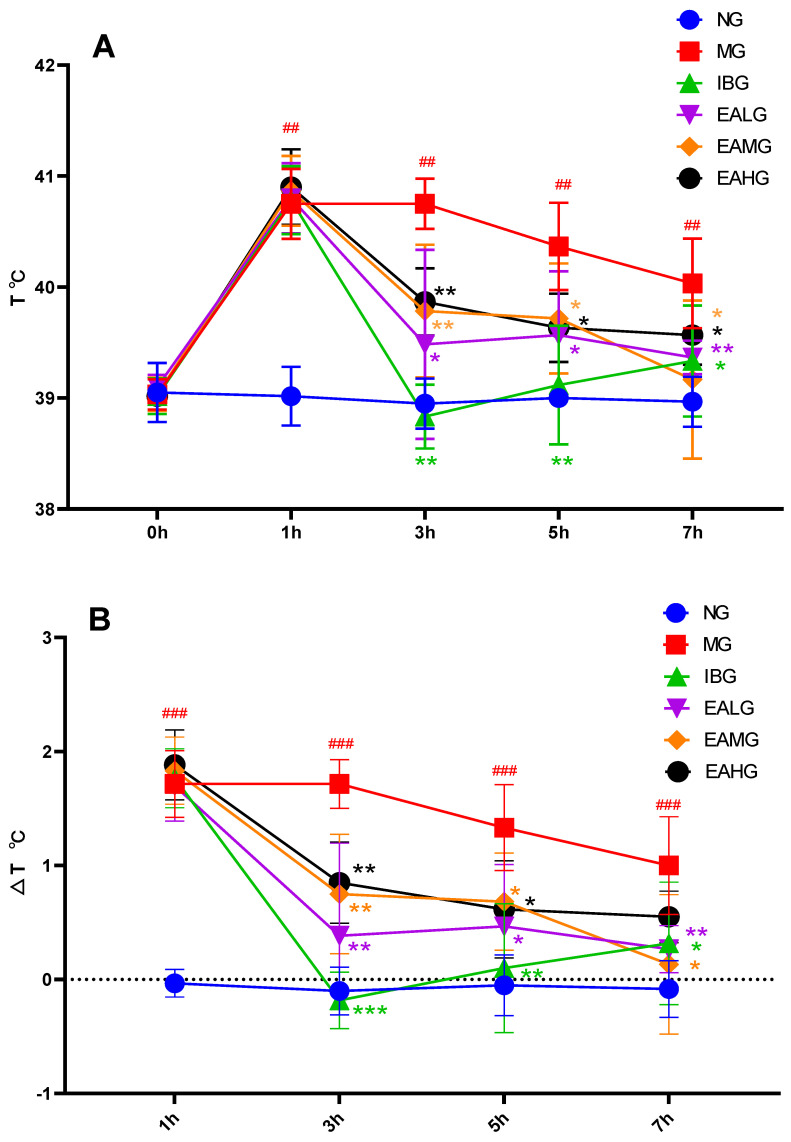
Antipyretic effects of ibuprofen and EA. (**A**): The rectal temperatures were measured at 0, 1, 3, 5, and 7 h after LPS administration (*n* = 6). (**B**): The rectal temperatures change value at 1, 3, 5, and 7 h after LPS administration (*n* = 6). *p* value is for individual time point. (^##^ *p* < 0.01 vs. NG,^###^ *p* < 0.001 vs. NG; * *p* < 0.05, ** *p* < 0.01, *** *p* < 0.001 vs. MG).

**Figure 2:**
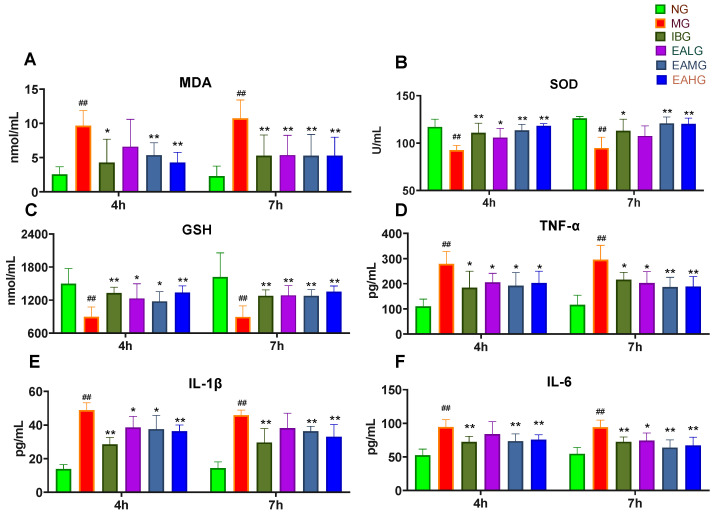
Anti-inflammatory effects of ibuprofen and EA at 4 h and 7 h after IPS administration (*n* = 6). (**A**): Effects of ibuprofen and EA in serum MDA secretion. (**B**): Effects of ibuprofen and EA in serum SOD activity. (**C**): Effects of ibuprofen and EA in serum GSH secretion. (**D**): Effects of ibuprofen and EA in serum TNF-α secretion. (**E**): Effects of ibuprofen and EA in serum IL-1β secretion. (**F**): Effects of ibuprofen and EA in serum IL-6 secretion. (^##^ *p* < 0.01 vs. NG; * *p* < 0.05, ** *p* < 0.01 vs. MG).

**Figure 3:**
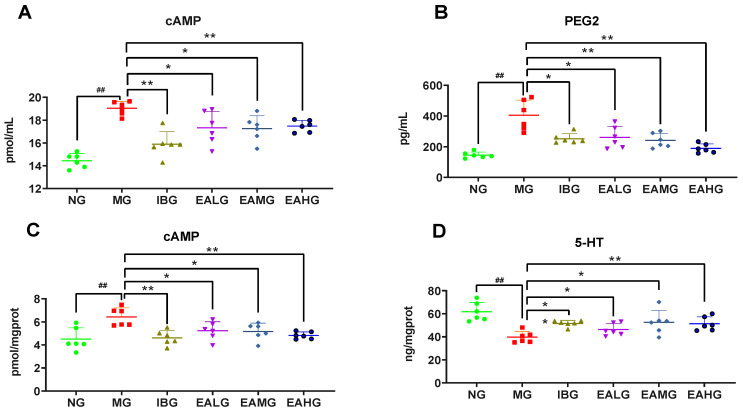
Anti-inflammatory effects of ibuprofen and EA (*n* = 6). (**A**): Effects of ibuprofen and EA on cerebrospinal fluid cAMP secretion. (**B**): Effects of ibuprofen and EA on cerebrospinal fluid PGE_2_ secretion. (**C**): Effects of ibuprofen and EA on hypothalamus cAMP secretion. (**D**): Effects of Ibuprofen and EA on hypothalamus 5-HT secretion. (^##^ *p* < 0.01 vs. NG; * *p* < 0.05, ** *p* < 0.01 vs. MG).

The data were analyzed using IBM SPSS Statistic 17.0 (IMB Corp., New York, NY, USA) and expressed as mean ± SEM. The data were statistically analyzed using a one-way ANOVA test. The LSD test was applied only if the ANOVA test was significant. Non-parametric data were statistically analyzed using the Kruskal–Wallis test. The Mann–Whitney U test was applied only if the Kruskal–Wallis test was significant. For Figures 1,2,3, 7 and 8, analyses were performed by using Prism 8 (GraphPad 8.00). *p* < 0.05 was regarded as statistically significant. The generated GC-MS raw data were converted from Proteowizard to mzML format. The online processing software (http://xcmsonline.scripps.edu (accessed on 28 October 2022)) XCMS) was used to extract, identify, align, and filter peaks. A dataset, including retention time, mass-to-charge ratio, and peak area, was obtained. After normalization by MS excel, the data were imported into SIMCA14.1 (Umea Sweden) for PCA and OPLS-DA for multivariate statistical analysis.

## 3. Results

### 3.1. Effects of EA on LPS-Induced Fever in Rabbits

First, we treated 30 rabbits with 4 μg/kg LPS to establish the fever model. The administration of LPS resulted in a significantly increased rectal temperature within 1 h compared to the normal group (NG) (*p* < 0.01), indicating that we successfully established the LPS-induced fever model (Figure 1A). Next, we divided the LPS-treated animals over five groups with six animals per group: a model group (MG); an ibuprofen-treated group (IBG, 20 mg/kg), which served as a positive control for the antipyretic effect; and three EA-treated groups, ellagic acid low dose group(EALG) (26 mg/kg), ellagic acid medium dose group(EAMG) (52 mg/kg), and ellagic acid high dose group(EAHG)(104 mg/kg). Compared to the MG group, the rectal temperatures of the IBG group were significantly lower at 3 h (*p* < 0.01) (Figure 1A) but showed a slow increasing tendency for the remaining duration of the experiment (Figure 1B), whereas the rectal temperatures of the EALG, EAMG, and EAHG groups were significantly lower at 3 h (*p* < 0.05 or *p* < 0.01) while keeping a downward trend for the remaining duration of the experiment (Figure 1A). Of note, throughout the experiment, the rectal temperatures observed in the EALG, EAMG, and EAHG groups were still above those observed in the NG group (Figure 1B).

### 3.2. Effect of EA on MDA, SOD, GSH, TNF-α, IL-1β, and IL-6 in Serum

Next, we analyzed the serum levels of inflammatory and antioxidant markers. For this, we collected blood at 4 and 7 h after LPS administration.

Compared to NG, both at 4 and 7 h, the levels of SOD and GSH were significantly decreased (*p* < 0.01), whereas the levels of MDA were significantly increased (*p* < 0.01) in serum of MG, respectively (Figure 2). Compared to MG, both at 4 and 7 h, the levels of SOD and GSH were significantly increased (*p* < 0.05 and *p* < 0.01), whereas the levels of MDA were significantly decreased (*p* < 0.05 or *p* < 0.01) in serum of IBG, EALG, EAMG, and EAHG, respectively. However, the level of SOD in EALG was not significantly increased (Figure 2).

Compared to NG, both at 4 and 7 h, the levels of IL-1β, IL-6, and TNF-α were significantly increased (*p* < 0.01) in serum of MG (Figure 2). Compared to MG, both at 4 and 7 h, the levels of IL-1β, IL-6, and TNF-α were significantly decreased (*p* < 0.05 or *p* < 0.01) in serum of IBG, EALG, EAMG, and EAHG (Figure 2).

### 3.3. Effect of EA on cAMP and PGE_2_ in Cerebrospinal Fluid

Next, we analyzed the levels of cAMP and PGE_2_ in cerebrospinal fluid. Compared to NG, the levels of cAMP and PGE_2_ were significantly increased (*p* < 0.01) in cerebrospinal fluid of MG. Compared to MG, the levels of cAMP and PGE_2_ were significantly decreased (*p* < 0.05 or *p* < 0.01) in cerebrospinal fluid of IBG, EALG, EAMG, and EAHG (Figure 3A,B).

### 3.4. Effect of EA on cAMP and 5-HT in Hypothalamus

Compared to NG, the level of cAMP was significantly increased (*p* < 0.01), whereas the level of 5-HT was significantly decreased (*p* < 0.01) in the hypothalamus of MG. Compared to MG, the level of cAMP in was significantly decreased (*p* < 0.05 or *p* < 0.01), whereas the level of 5-HT was significantly increased (*p* < 0.05 or *p* < 0.01) in the hypothalamus of IBG, EALG, EAMG, and EAHG (Figure 3C,D).

### 3.5. Effect of EA on p-NF-κB p65 and COX-2 Expression in Hypothalamus

**Figure 4:**
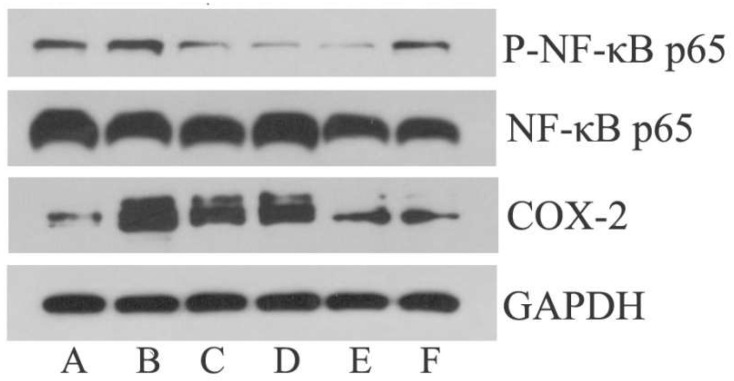
Effect of IBG and EA on p-NF-κB p65 and COX-2 expression in hypothalamus of LPS-treated rabbits. A~F: NG, MG, IBG, EALG, EAMG, EAHG.

Compared to NG, the protein levels of p-NF-κB p65 and COX-2 were significantly up-regulated (*p* < 0.05 or *p* < 0.01) in MG (Figure 4 and Table 1). Compared to MG, the protein levels of p-NF-κB p65 were significantly down-regulated (*p* < 0.05) in IBG, EALG, EAMG, and EAHG (Figure 4 and Table 1). Compared to MG, the protein levels of COX2 were significantly down-regulated (*p* < 0.05 or *p* < 0.01) in IBG, EAMG, and EAHG but not in EALG (Figure 4 and Table 1).

### 3.6. Serum Metabolomics Profile and Multivariate Data Analysis

#### 3.6.1. Principal Component Analysis (PCA)

##### PCA Analysis of Serum Samples Collected at 4 h

**Figure 5:**
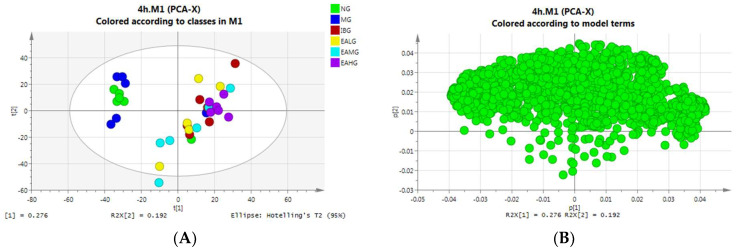

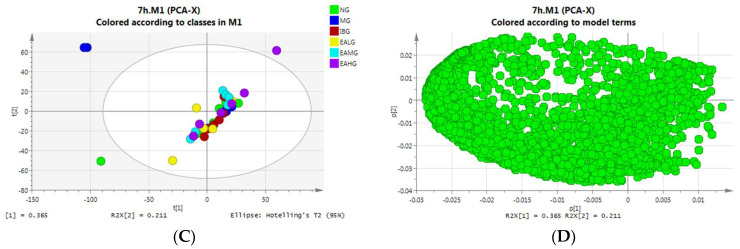
PCA analysis. (**A**)**:** The distribution score of 4 h serum samples among six groups. (**B**)**:** The serum sample distribution load diagram at 4 h. (**C**)**:** The distribution score of 7 h serum samples among six groups. (**D**)**:** The serum sample distribution load diagram at 7 h.

In the next step, we used GC-MS to analyze the metabolite profile in the serum of the different groups. Then, we applied PCA analysis—an approach for dimensionality reduction that identifies a set of orthogonal axes, called principal components (PCs), that capture the maximum variance in the data—to visually analyze the similarities and differences between the experimental groups. Based on the PCA plot, we show that the separation between the six groups at 4 h after LPS administration is not very good. This suggests that LPS-induced fever has a dominant effect on the level of endogenous metabolites in the serum of rabbits, which consequently results in overlapping metabolite profiles between the groups (Figure 5A). In the next step, we constructed a loading diagram of our PCA analysis. The PCA loadings are the coefficients of the linear combination of the original variables from which the PCs are constructed. In other words, the coordinates of each variable correspond to the correlation and directivity with PC1 and PC2, respectively. As such, R^2^X(1) and R^2^X(2) represent the corresponding contribution rates of PC1 and PC2, respectively. The PCA loading diagram analysis indicated that the contribution of the two principal components is greater (R^2^X(1) = 0.276, R^2^X(2) = 0.192) (Figure 5B).

##### PCA Analysis of Serum Samples Collected at 7 h

At 7 h after LPS administration, the separation between the six groups was again not very good, while some samples deviated from the model range (Figure 5C). We speculate that the LPS-induced fever caused individual animal-specific differences between the endogenous metabolites. The PCA loading diagram analysis indicated that the contribution of the two principal components is greater (R^2^X(1) = 0.365, R^2^X(2) = 0.211) (Figure 5D).

#### 3.6.2. Orthogonal Partial Least Squares–Discriminant Analysis (OPLS-DA)

##### OPLS-DA Analysis of Serum Samples Collected at 4 h

**Figure 6:**
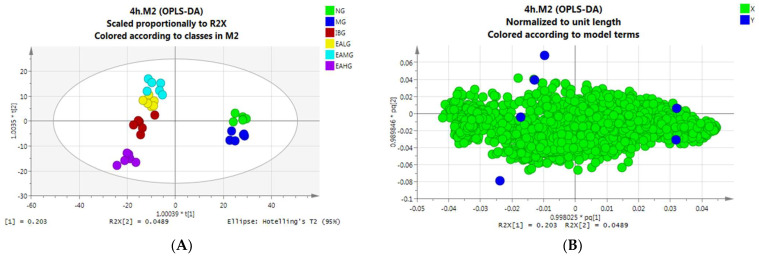

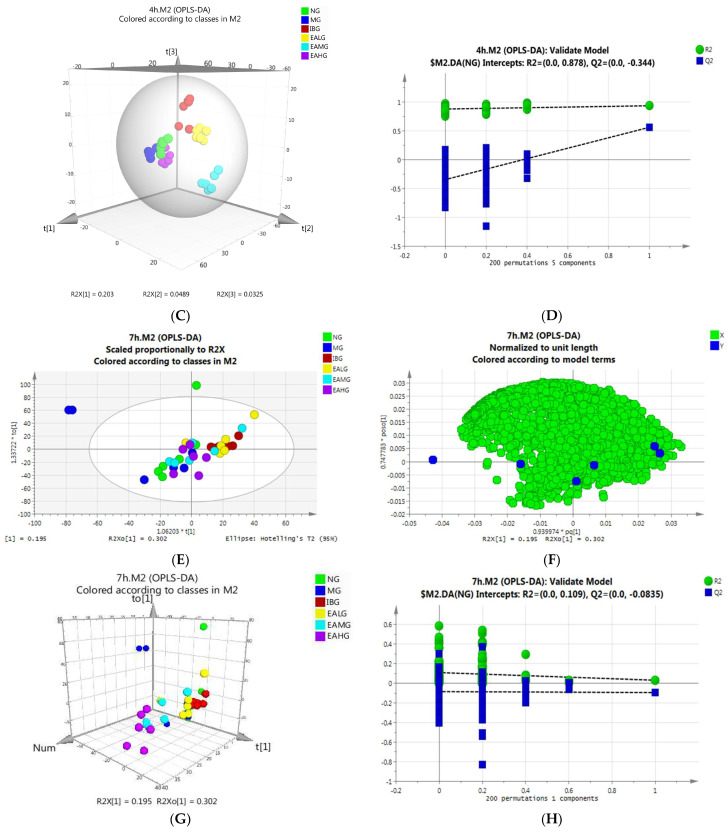
OPLS-DA analysis results. (**A**): The distribution score of 4 h serum samples among six groups. (**B**): 4 h serum sample distribution load diagram. (**C**): 3D score chart of 4 h serum sample. (**D**): 4 h serum sample random displacement test (*n* = 200) and model interpretation rate. (**E**): The distribution score of 7 h serum samples among 6 groups. (**F**): 7 h serum sample distribution load diagram. (**G**): 3D score chart of 7 h serum sample. (**H**): 7 h serum sample random displacement test (*n* = 200) and model interpretation rate.

Following PCA, we performed OPLS-DA analysis to gain more insight into our obtained dataset. In our established OPLS-DA model, the parameters such as random permutation test (*n* = 200), interpretation rate (R^2^), and prediction ability (Q^2^) were evaluated. As such, we obtained a good separation between the six groups (Figure 6A). A load diagram can identify differences in metabolites between groups. Each point in the diagram represents a metabolite. The greater the distance from the axis, the greater the contribution to the difference between the groups. The contribution rates of PC1 and PC2 are, respectively, R^2^X(1) = 0.203,R^2^X(2) = 0.0489 (Figure 6B). We can see from the 3D figure that the six groups of samples have good separation in the spatial dimension (Figure 6C), with R^2^ = 0.878 and Q^2^ = −0.344 indicating that the model has good stability and prediction ability (Figure 6D).

##### OPLS-DA Analysis of Serum Samples Collected at 7 h

At 7 h after LPS administration, the samples between the six groups overlapped, while some samples even exceeded the model range (Figure 6E). Similar to the PCA analysis, we speculate that the LPS-induced fever caused individual animal-specific differences between the endogenous metabolites. In our established OPLS-DA model, the parameters such as random permutation test (*n* = 200), interpretation rate (R^2^), and prediction ability (Q^2^) were evaluated. As such, we obtained a good separation between the six groups with R^2^ = 0.109 and Q^2^ = −0.0835, again indicating that the model has good stability and prediction ability (Figure 6H). In addition, we see that the contribution rates of PC1 and PC2 are, respectively, R^2^X(1) = 0.302, R^2^X(2) = 0.195 (Figure 6F). A 3D diagram shows that there is good separation between the samples, but the aggregation between the groups is not very good (Figure 6G).

#### 3.6.3. Identification of Potential Biomarkers

In the following step, based on the data obtained with the OPLS-DA model and subsequent score and load plots, we screened for potential metabolites with large contribution scores. Additionally, while using VIP > 1 and *p* < 0.05 as screening criteria, we compared the corresponding retention time and characteristic mass-to-charge ratio with the national Institute of Standards and Technology(NIST) library (match score > 80%).

**Figure 7:**
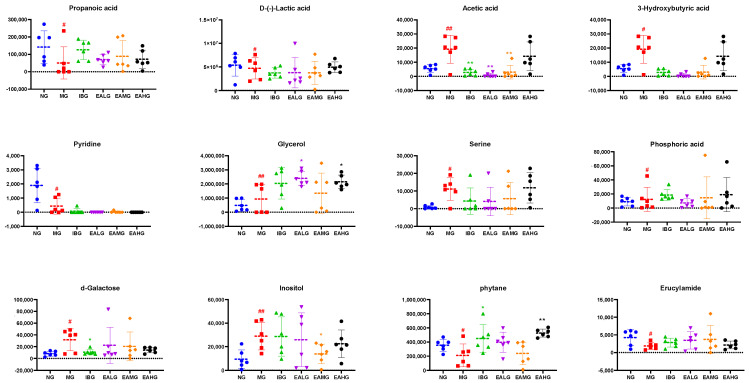
Scatter plot of 12 differential metabolites in serum within 4 h. ^#^ *p* < 0.05, ^##^ *p* < 0.01 vs. NG; * *p* < 0.05, ** *p* < 0.01 vs. MG (*n* = 6).

As such, we identified 12 metabolites, which were differentially regulated at 4 h after LPS administration in the MG group compared to NG (Table 2). From these, four metabolites had lower levels, while eight had higher levels in the LPS-treated animals compared to NG. Interestingly, compared to MG, seven metabolites—including propanoic acid, 3-hydroxybutyric acid, pyridine, glycerol, phosphoric acid, phytane, erucylamide—were significantly different in the EA-treated groups (*p* < 0.05) (Figure 7).

**Figure 8:**
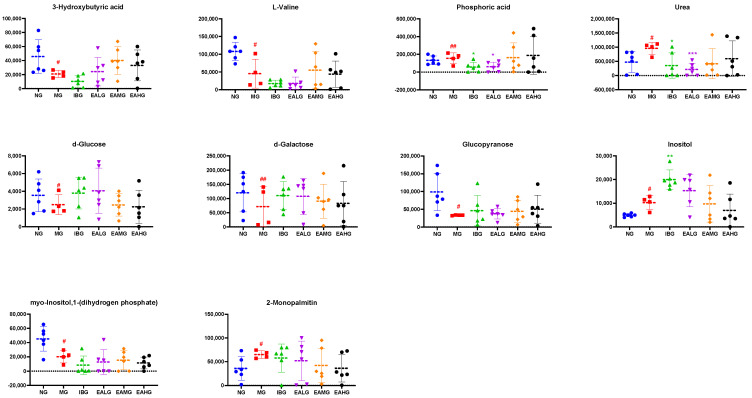
Scatter plot of 10 differential metabolites in serum within 7 h. ^#^ *p* < 0.05, ^##^ *p* < 0.01 vs. NG; * *p* < 0.05, ** *p* < 0.01, *** *p* < 0.001 vs. M. (*n* = 6).

Further, we identified 10 metabolites, which were differentially regulated at 7 h after LPS administration in the MG group compared to NG (Table 3). From these, six metabolites had lower levels, while four had higher levels in the LPS-treated animals compared to NG. Interestingly, compared to MG, six metabolites—including 3-hydroxybutyric acid, urea, D-galactose, glucopyranose, inositol, and 2-monopalmitin—were significantly different in the EA-treated groups (*p* < 0.05) (Figure 8).

Combined, considering that both at 4 and 7 h 3-hydroxybutyric acid, phosphoric acid, D-galactose, and inositol were identified as differential metabolites in EA-treated groups compared to MG suggests that the antipyretic effect of EA might involve the regulation of these four metabolites.

#### 3.6.4. Metabolic Pathway Analysis of the Potential Biomarkers

**Figure 9:**
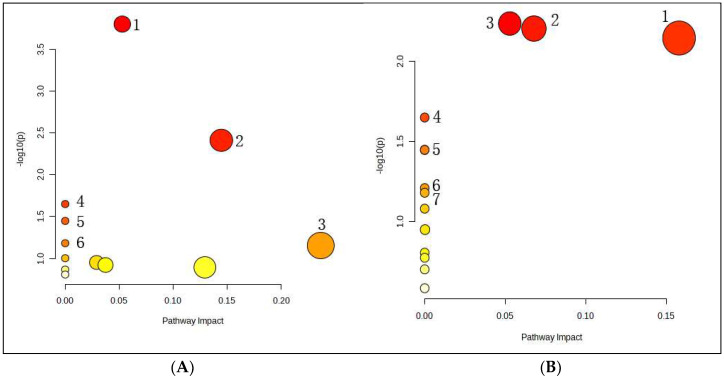
(**A**): Analysis of serum metabolic pathways at 4 h after EA administration: (1) galactose metabolism; (2) pyruvate metabolism; (3) glycerol metabolism; (4) synthesis and degradation of ketone bodies; (5) ascorbic acid and aldehyde acid metabolism; (6) butyric acid metabolism. (**B**): Analysis of serum metabolic pathways at 7 h after EA administration: (1) galactose metabolism; (2) phosphatidylinositol signal system; (3) inositol phosphate metabolism; (4) synthesis and degradation of ketone bodies; (5) ascorbic acid and aldehyde acid metabolism; (6) biosynthesis of valine, leucine, and isoleucine; (7) Arginine biosynthesis.

In order to gain a better insight into the metabolic cascades related to fever, we screened differential metabolites with MetaboAnalyst 4.0 (http://www.metaboanalyst.ca/ (accessed on 21 November 2022)) for metabolic pathway analysis. Four hours after EA administration to LPS-treated animals, we found six significantly enriched pathways: galactose metabolism, pyruvate metabolism, glycerol metabolism, synthesis and degradation of ketone bodies, ascorbic acid and aldehyde acid metabolism, and butyric acid metabolism. This involved five potential biomarkers, such as D-(-)-lactic acid, acetic acid, glycerol, D-galactose, and inositol (Figure 9). Seven hours after EA administration to LPS-treated animals, we found six significantly enriched pathways: galactose metabolism; phosphatidylinositol signal system; inositol phosphate metabolism; synthesis and degradation of ketone bodies; ascorbic acid and aldehyde acid metabolism; biosynthesis of valine, leucine, and isoleucine; and arginine biosynthesis, which involved six metabolites, such as 3-hydroxybutyric acid, L-valine, phosphoric acid, urea, D-galactose, and inositol, myo-Inositol 1-(dihydrogen phosphate) (Figure 9). These observations suggest that EA may exert its antipyretic effects by regulating energy and fat metabolism, among other pathways.

#### 3.6.5. Correlation Analysis between Metabolic and Inflammatory Biomarkers

In a final step, we characterized the relationship between the identified metabolic biomarkers and the inflammatory indicators with Pearson correlation. Four hours after EA administration to LPS-treated animals, we found that six metabolites significantly correlated with four inflammatory markers: propionic acid correlated negatively with MDA and TNF-α (*p* < 0.05) and positively with GSH (*p* < 0.05), respectively; acetic acid correlated negatively with GSH (*p* < 0.05); pyridine correlated negatively with IL-1β (*p* < 0.01) while having a moderate correlation; and serine, D-galactose, and inositol correlated positively with IL-1β (*p* < 0.05) (Table 4). Seven hours after EA administration to LPS-treated animals, we found that five metabolites significantly correlated with five inflammatory markers: L-valine moderately correlated positively with GSH (*p* < 0.01) and correlated negatively with MDA, IL-1β, and TNF-α (*p* < 0.01and *p* < 0.05); urea correlated negatively with SOD (*p* < 0.05); inositol correlated negatively with SOD and GSH (*p* < 0.01 and *p* < 0.05), and positively with IL-1β (*p* < 0.01); myo-inositol, 1-(dihydrogen phosphate) correlated positively with GSH (*p* < 0.01) and negatively with MDA, IL-1β, and TNF-α (*p* < 0.01 and *p* < 0.05); and 2-monopalmitin correlated positively with MDA, SOD, IL-1β, and TNF-α (*p* < 0.01) and negatively with SOD (*p* < 0.01) (Table 5). These results indicate the biological significance of the selected metabolites and prove that EA plays an antipyretic role by regulating inflammatory factors and differential metabolites.

## 4. Discussion

The onset of fever is an inflammation-mediated body injury process that closely resembles the innate immune response induced by exposure to LPS. The regulation of body temperature requires a delicate balance between heat production and heat dissipation, which is regulated by the hypothalamus [39]. Considering that, among the different animal models of fever, LPS-induced pyrexia in rabbits is one of the most widely used, we selected rabbits for our model as they more easily develop fever than rats and have a docile nature.

In this study, we first monitored the temperature and its changing value in pyretic rabbits. Then, we measured increasing concentrations of the inflammatory factors MDA, IL-6, TNF-α, and IL-6 in serum and cAMP, PGE_2_, and 5-HT in cerebrospinal fluid and hypothalamus of LPS-treated animals (MG group). After the administration of EA, the rectal temperature of rabbits was significantly decreased within 2 h, and the levels of IL-1β, IL-6, and TNF-α were significantly decreased within 3 h. After continuous administration for 6 h, EA affected the thermoregulation center through the blood–brain barrier, inhibited the secretion of cAMP, PGE_2_, and 5-HT in cerebrospinal fluid and hypothalamus, and reduced the rectal temperature of rabbits. It is suggested that EA plays an antipyretic role by reducing central heat mediators and inflammatory factors, and the duration of action is more than 6 h.

Among others, NF-κB is involved in regulating the cell cycle and apoptosis, cell adhesion and migration, inflammatory response, and immune regulation [40]. Activation of NF-κB mainly occurs via two pathways: the classical pathway (activation of NF-κB with P50 and P65 structure) and a side pathway (activation of NF-κB with P100 or P105 structure) [41]. In the classical pathway, external stimuli-mediated phosphorylation of IKKβ, a subunit of the IKK kinase, results in the phosphorylation and ubiquitin-mediated degradation of IKB-α and subsequent release of the P50/P65 isodimer [42].

Previous studies indicated that extracellular signal-regulated protein kinases, such as NF-κB, are key components in response to various external stress inducers and are able to induce the production of inflammatory factors TNF-α, IL-1β, and IL-6 while stimulating the release of cytokines and chemokines [25,26,27,28].

A correlation between NF-κB and COX-2 expression was previously shown, resulting in the identification of two NF-κB binding sites in the 5′ flanking region of the COX-2 gene and the speculation that the expression of the COX-2 gene is regulated by NF-κB [43]. As PGE_2_ was shown to have no interaction with activators associated with NF-κB activation, it was suggested that activation of COX-2 may also regulate NF-κB through downstream products [44]. Recent studies indicated that EA can block the induction of TNF-α by inhibiting the NF-κB and COX-2 pathways [30]. Furthermore, in the study of anti-inflammation and anti-nephrotoxicity, EA can reduce the secretion of IL-1β and TNF-α inflammatory factors, promote the increase in IL-10 anti-inflammatory factors, reduce the expression of COX-2 and NF-κB, and has anti-oxidative stress effect [30,38]. In addition, EA was shown to have a protective effect on LPS-induced cognitive impairment, potentially through the inhibition of NF-κB and COX-2 pathways and subsequent reduction in oxidative damage, improvement in synaptic plasticity, and restoration of cholinergic function [45].

In our study, we found increased levels of p-NF-κB P65 and COX-2 in the hypothalamus of our LPS-induced fever model. Interestingly, EA treatment decreased the p-NF-κB P65 and COX-2 levels, suggesting that EA might stimulate NF-κB signaling, as such exerting a protective effect against LPS-mediated oxidant damage through the inhibition of NF-κB/COX-2 signaling and subsequent inflammation suppression.

**Figure 10:**
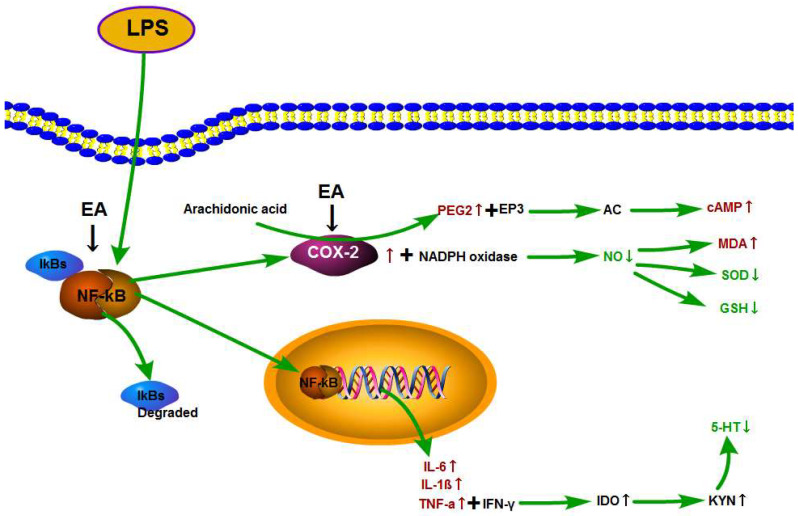
EA plays an antipyretic and anti-inflammatory role through regulation of the NF-κB/COX-2 pathway and subsequent inhibition of inflammatory factors.

In Figure 10, we present a graphical overview of our LPS-induced fever model and the potential antipyretic action of EA. First, the administration of LPS results in the phosphorylation and degradation of IκBα and the subsequent release of the P50/P65 heterodimer, which translocates to the nucleus, where it promotes the expression of the inflammatory factors IL-1β, TNF-α, and IL-6. High levels of TNF-α and interferon gamma (IFN-γ) activate and induce the activation of indoleamine-2, 3-dioxygenase (IDO) and increase the levels of kynurenine (KYN). High levels of KYN consequently result in decreased free tryptophan levels, which naturally limits the total amount of the latter which is able to pass through the blood–brain barrier. Considering that tryptophan is the precursor of 5-HT synthesis in the brain, low levels of tryptophan in the brain directly affect the synthesis and release of 5-HT [46,47].

COX-2-mediated NF-κB-induced activation of COX-2 promotes the expression of its downstream product, PGE_2_. Subsequently, PGE_2_ binds to the transmembrane receptor EP3 on the cell membrane, which couples G protein and blocks the activation of adenyl cyclase (AC), resulting in limited amounts of ATP-derived cAMP [48,49]. In parallel, COX-2 and NADPH oxidase synergistically reduce the bioavailability of NO, which promotes the accumulation of reactive oxygen species and reduces the activities of SOD and GSH, resulting in an aggravated oxidative stress reaction through increased MDA levels. Previously, a correlation between oxidative stress and inflammation has been demonstrated [50].

Based on our results, we suggest that EA might reduce the production of IL-1β, TNF-α, and IL-6 through the inhibition of IKB-α expression and subsequent translocation of NF-κB P65 from the cytoplasm to the nucleus. Moreover, EA-mediated inhibition of COX-2 might reduce the level of PEG2 and, as such, relieve the oxidative stress reaction. Taken together, these results suggest that the antipyretic and anti-inflammatory effects of EA might be realized through the inhibition of the NF-κB/COX-2 signaling pathway.

Next, we analyzed the effects of EA on the changes in the serum metabolites in rabbits with LPS-induced fever at 4 and 7 h. In the serum of LPS-treated animals (MG group) collected 4 h after LPS administration, we identified 12 differential metabolites. Interestingly, the LPS-induced effect on 7 of those 12 metabolites was reversed by subsequent EA administration. Based on the correlation coefficient values with the inflammatory markers, we identified propionic acid and pyridine as two key metabolites. In the serum of LPS-treated animals (MG group) collected 7 h after LPS administration, we identified ten differential metabolites. Similarly, the LPS-induced effect on six of those ten metabolites was reversed by subsequent EA administration. Based on the correlation coefficient values with the inflammatory markers, we identified L-valine, inositol, urea, and 2-palmitic acid monoglyceride as key metabolites.

**Figure 11:**
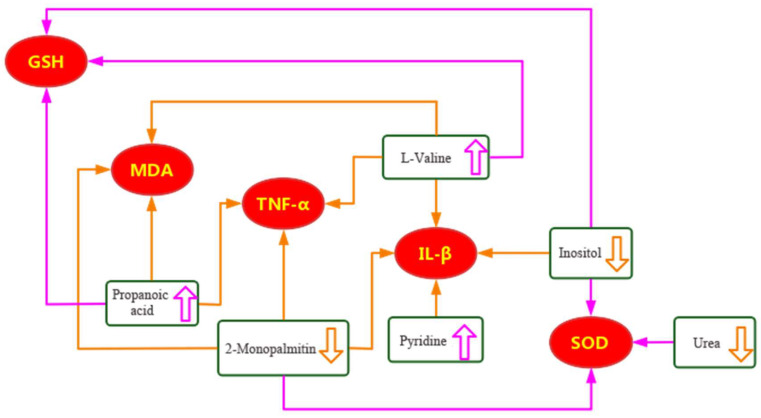
Suggested mechanism of how the EA-induced antipyretic and anti-inflammatory effect operates through the regulation of six differential metabolites resulting in the inhibition of inflammatory factors. The green box indicates a differential metabolite; the red oval is an inflammatory factor; orange line indicates down-regulation; purple line indicates up-regulation effect; the thick arrows indicates the up and down of metabolite levels; the thin arrows indicates the interaction between inflammatory factors and metabolites.

Based on the relationship between six differential metabolites and the inflammatory markers MDA, SOD, GSH, IL-1β, and TNF-α, we suggest the following mechanism for EA-induced reduced inflammation and the limitation of the oxidative stress state (Figure 11). On the one hand, EA up-regulated the levels of propionic acid, pyridine, and L-valine, which results in lower levels of MDA, IL-1β, and TNF-α while increasing the activity of GSH. On the other hand, EA down-regulated the levels of inositol, urea, and 2-monopalmitin, which equally results in lower levels of MDA, IL-1β, and TNF-α while increasing the activity of SOD and GSH.

In addition, based on the pathway analysis, we suggest that the pathways of the galactose metabolism, synthesis and degradation of ketone bodies, and ascorbic acid and aldehyde acid metabolism are closely related to the antipyretic and anti-inflammatory effects of EA through their effect on D-galactose and inositol, further suggesting that EA exerts its antipyretic and anti-inflammatory effects through the regulation of specific metabolites. Moreover, considering that the pathways of the pyruvate metabolism, glycerol metabolism, inositol phosphate metabolism, as well as the biosynthesis of valine, leucine, and isoleucine, all involve energy substances such as sugar, fat, and amino acids, we suggest that the antipyretic effect of EA is, among others, related to the regulation of the energy and fat metabolism.

Based on the correlation results between the levels of the differential metabolites and inflammatory markers in serum, we found that ten differential metabolites significantly correlated with MDA, SOD, GSH, IL-1β, and TNF-α (*p* < 0.05). The fact that the levels of propionic acid, pyridine, L-valine, inositol, urea, and 2-monopalmitin positively correlated with the levels of these five inflammatory markers further proved that EA might exert its antipyretic effect through the regulation of differential metabolites and inflammatory factors.

The current study has limitations. First, whereas EA was administered in three different dose groups, all groups had similar rectal temperatures and comparable MDA, GSH, and TNF-α levels. We suggest that the optimal effective dose of EA in order to have antipyretic and anti-inflammatory effects should be below 26 mg/kg. This hypothesis should be verified in future studies by applying lower EA doses. Second, whereas we identified 12 and 10 differential metabolites in the 4 h and 7 h metabolite analysis, only 4 of them were similar. It is suggested that during the process of fever, the function of rabbits may be affected by the interplay between LPS, EA, and various secretory factors, ultimately resulting in metabolite differences. This should be addressed in future studies.

## 5. Conclusions

In summary, our study established the relationship between EA and various inflammatory markers, such as TNF-α, IL-6, IL-1β, PGE_2_, and cAMP, and clarified the mechanism of the COX-2/NF-κB signaling pathway. Combined with the metabolomics analysis, our study revealed the effects of EA on multiple endogenous biomarkers, reflecting the characteristics of a multi-component, multi-target, and multi-pathway mechanism. Overall, based on these data, our study provides a scientific basis of how EA acts as an anti-inflammatory agent in the treatment of fever in a rabbit animal model.

## Figures and Tables

**Table 1:** Effect of EA on p-NF-κB p65 and COX-2 in hypothalamus.

Groups (Dose)	p-NF-κB p65/GAPDH	COX-2/GAPDH
NG	0.29 ± 0.06	0.39 ± 0.1
MG	0.54 ± 0.06 ^##^	1.44 ± 0.37 ^#^
IBG (20 mg/kg)	0.28 ± 0.1 ^▲^	0.74 ± 0.36
EALG (26 mg/kg)	0.24 ± 0.14 ^▲^	1.11 ± 0.23
EAMG(52 mg/kg)	0.18 ± 0.11 ^▲^	0.53 ± 0.15 ^▲^
EAHG(104 mg/kg)	0.29 ± 0.06 ^▲▲^	0.39 ± 0.16 ^▲^

Note: $\bar{x}$ *± s*, *n* = 3, ^#^ *p* < 0.05, ^##^ *p* < 0.01, compared to NG. ^▲^ *p* < 0.05, ^▲▲^ *p* < 0.01, compared to MG.

**Table 2:** The information and change trend of differential metabolites among six groups of samples within 4 h (*n* = 6).

HMDB	t_R_/min	Metabolite	Formula	m/z	KEGG	*p*	Match Score %	NG vs. MG	MG vs. IBG	MG vs. EALG	MG vs. EAMG	MG vs. EAHG
HMDB0000237	5.60	Propanoic acid	C_3_H_6_O_2_	28,29,74	C00163	0.028	80.01	↓	↑	↑	↑	↑
HMDB0001311	5.92	D-(-)-Lactic acid	C3H6O3	147,117,191	C00256	0.043	94.4	↑	↑	↑	↑	↑
HMDB0000042	6.30	Acetic acid	C2H4O2	43,45,60	C00033	0.024	86.59	↑	↓	↑	↑	↑
HMDB0000357	7.41	3-Hydroxybutyric acid	C_4_H_8_O_3_	147,117,191	C01089	0.043	94.87	↑	↓	↓	↓	↑
HMDB0000926	7.71	Pyridine	C5H5N	79,52,40	C00747	0.038	82.1	↓	↑	↑	↑	↑
HMDB0000131	8.88	Glycerol	C3H8O3	205,117,103	C00116	0.006	96.25	↑	↑	↓	↓	↓
HMDB0062263	9.74	Serine	C3H7NO3	204,218,100	C00716	0.037	89.75	↑	↓	↑	↑	↑
HMDB0002142	10.42	Phosphoric acid	H3PO4	299,300,133	C00009	0.031	93.28	↑	↓	↑	↓	↓
HMDB0000143	17.93	d-Galactose	C6H12O6	147,205,103	C00984	0.014	88.64	↑	↓	↑	↑	↑
HMDB0000211	20.14	Inositol	C6H12O6	147,217,305	C00137	0.008	91.71	↑	↓	↑	↑	↑
NA	22.23	phytane	C20 H42	57,71,43	NA	0.018	82.72	↓	↑	↑	↑	↓
NA	29.71	Erucylamide	C22H43NO	59,72,55	NA	0.042	84.56	↓	↑	↓	↑	↓

The *p* value was normal group vs. model group. ↑: level up; ↓: level down.

**Table 3:** The information and change trend of differential metabolites among six groups of samples within 7 h (*n* = 6).

HMDB	t_R_/min	Metabolite	Formula	m/z	KEGG	*p*	Match Score %	NG vs. MG	MG vs. IBG	MG vs. EALG	MG vs. EAMG	MG vs. EAHG
HMDB0000357	7.57	3-Hydroxybutyric acid	C_4_H_8_O_3_	147,117,191	C01089	0.029	94.19	↓	↓	↑	↑	↑
HMDB0000883	8.41	L-Valine	C5H11NO2	146,156,130	C00183	0.045	93.42	↓	↓	↓	↑	↓
HMDB0002142	10.43	Phosphoric acid	H_3_PO_4_	299,300,133	C00009	0.001	92.77	↑	↓	↓	↑	↑
HMDB0000294	11.59	Urea	CH4N2O	60,44,17	C00086	0.030	92.61	↑	↓	↓	↓	↓
HMDB0000122	18.00	d-Glucose	C_6_H_12_O_6_	217,218,133	C00221	0.035	90.2	↓	↑	↑	↓	↓
HMDB0000143	18.26	d-Galactose	C_6_H_12_O_6_	147,205,103	C00984	0.005	92.88	↓	↑	↑	↑	↑
NA	19.07	Glucopyranose	C_6_H_12_O_6_	217,218,133	NA	0.020	95.95	↓	↑	↑	↑	↑
HMDB0000211	20.29	Inositol	C_6_H_12_O_6_	147,217,305	C00137	0.035	94.12	↑	↑	↑	↓	↓
HMDB0000213	24.13	myo-Inositol,1-(dihydrogen phosphate)	C6H13O9P	73,147,271	C01177	0.017	85.35	↓	↓	↓	↓	↓
HMDB0011533	25.25	2-Monopalmitin	C19H38O4	129,103,147	NA	0.026	80.76	↑	↓	↓	↓	↓

The *p* value was normal group vs. model group. ↑: level up; ↓: level down.

**Table 4:** Pearson correlation coefficient between serum metabolites and inflammatory markers at 4 h.

Metabolite	The Correlation Coefficient: r					
	MDA	SOD	GSH	IL-1β	IL-6	TNF-α
Propanoic acid	−0.391 *	+0.153	+0.422 *	−0.319	−0.280	−0.330 *
D-(-)-Lactic acid	−0.101	+0.102	+0.013	−0.106	+0.058	+0.090
Acetic acid	+0.247	−0.304	−0.332 *	+0.318	+0.253	+0.168
3-Hydroxybutyric acid	−0.128	+0.095	+0.088	+0.068	−0.054	+0.134
Pyridine	−0.266	+0.225	+0.237	−0.502 **	−0.388	−0.359
glycerol	+0.119	+0.005	+0.074	+0.235	+0.187	+0.250
Serine	+0.014	−0.138	−0.136	+0.394 *	+0.290	+0.304
Phosphoric acid	−0.178	+0.123	−0.052	+0.033	+0.120	+0.022
d-Galactose	+0.147	−0.314	−0.325	+0.367 *	+0.361	+0.331
Inositol	−0.029	−0.179	−0.105	+0.360 *	+0.523	+0.247
phytane	−0.307	+0.255	+0.215	−0.154	−0.160	−0.084
Erucylamide	+0.007	+0.079	+0.089	−0.150	−0.316	−0.128

“+” positive correlation; “−” negative correlation; ** *p* < 0.01; * *p* < 0.05.

**Table 5:** Pearson correlation coefficient between serum metabolites and inflammatory markers at 7 h.

Metabolite	The Correlation Coefficient: r					
	MDA	SOD	GSH	IL-1β	IL-6	TNF-α
3-Hydroxybutyric acid	−0.046	+0.316	+0.247	−0.263	−0.369	−0.471
L-Valine	−0.339 *	+0.284	+0.513 **	−0.501 **	−0.515	−0.463 *
Phosphoric acid	−0.242	+0.015	+0.012	+0.03	−0.098	−0.13
Urea	−0.032	−0.354 *	−0.208	+0.081	+0.156	+0.211
d-Glucose	−0.039	+0.027	−0.059	−0.037	+0.144	−0.091
d-Galactose	−0.14	+0.061	+0.249	−0.181	−0.034	−0.009
Glucopyranose	−0.414	+0.38	+0.297	−0.577	−0.313	−0.428
Inositol	+0.188	−0.249 *	−0.336 **	+0.293 **	0.387	+0.278
myo-Inositol,1-(dihydrogen phosphate)	−0.388 *	+0.153	+0.395 **	−0.531 **	−0.347	−0.445 *
2-Monopalmitin	+0.232 **	−0.291 **	−0.397	+0.353 **	+0.29	+0.261 **

“+” positive correlation; “−” negative correlation; ** *p* < 0.01; * *p* < 0.05.

## Data Availability

The raw data supporting the conclusions of this article will be made available by the authors on request.
